# Patients and Professionals as Partners in Hypertension Care: Qualitative Substudy of a Randomized Controlled Trial Using an Interactive Web-Based System Via Mobile Phone

**DOI:** 10.2196/26143

**Published:** 2021-06-03

**Authors:** Ulrika Andersson, Ulrika Bengtsson, Agneta Ranerup, Patrik Midlöv, Karin Kjellgren

**Affiliations:** 1 Department of Clinical Sciences Malmö Lund University Malmö Sweden; 2 Institute of Health and Care Sciences University of Gothenburg Gothenburg Sweden; 3 University of Gothenburg Centre for Person-Centred Care University of Gothenburg Gothenburg Sweden; 4 Department of Applied Information Technology University of Gothenburg Gothenburg Sweden; 5 Department of Health, Medicine and Caring Sciences Linköping University Linköping Sweden

**Keywords:** eHealth, digital health, hypertension, mobile phones, patient-professional partnership, person-centered care, self-management

## Abstract

**Background:**

The use of technology has the potential to support the patient´s active participation regarding treatment of hypertension. This might lead to changes in the roles of the patient and health care professional and affect the partnership between them.

**Objective:**

The aim of this qualitative study was to explore the partnership between patients and health care professionals and the roles of patients and professionals in hypertension management when using an interactive web-based system for self-management of hypertension via the patient’s own mobile phone.

**Methods:**

Focus group interviews were conducted with 22 patients and 15 professionals participating in a randomized controlled trial in Sweden aimed at lowering blood pressure (BP) using an interactive web-based system via mobile phones. The interviews were audiorecorded and transcribed and analyzed using thematic analysis.

**Results:**

Three themes were identified: the technology, the patient, and the professional. The technology enabled documentation of BP treatment, mainly for sharing knowledge between the patient and the professional. The patients gained increased knowledge of BP values and their relation to daily activities and treatment. They were able to narrate about their BP treatment and take a greater responsibility, inspired by new insights and motivation for lifestyle changes. Based on the patient’s understanding of hypertension, professionals could use the system as an educational tool and some found new ways of communicating BP treatment with patients. Some reservations were raised about using the system, that it might be too time-consuming to function in clinical practice and that too much measuring could result in stress for the patient and an increased workload for the professionals. In addition, not all professionals and patients had adopted the instructions regarding the use of the system, resulting in less realization of its potential.

**Conclusions:**

The use of the system led to the patients taking on a more active role in their BP treatment, becoming more of an expert of their BP. When using the system as intended, the professionals experienced it as a useful resource for communication regarding BP and lifestyle. Patients and professionals described a consultation on more equal grounds. The use of technology in hypertension management can promote a constructive and person-centered partnership between patient and professional. However, implementation of a new way of working should bring benefits and not be considered a burden for the professionals. To establish a successful partnership, both the patient and the professional need to be motivated toward a new way of working.

**Trial Registration:**

ClinicalTrials.gov NCT03554382; https://clinicaltrials.gov/ct2/show/NCT03554382

## Introduction

### Background

Medical advances and better living conditions have led to increasing lifespans and a growing population living with chronic conditions such as hypertension [1]. With limited health care resources, new, more effective ways of managing chronic conditions need to be developed [2]. Patients cannot be regarded as passive recipients of care but will need to be the main providers of care for themselves. With this, the role of health care professionals will also need to change from being the expert provider of care to being a cocreator of care with the patient [3,4]. During 2020 and the COVID-19 pandemic, the need for this has become even more evident. Patients need to be able to perform effective self-management in their homes and not be dependent on visiting or using health care facilities [5]. However, self-managing high blood pressure (BP) is something patients do every day by choosing what to eat, deciding whether to exercise, trying to decrease stress, and remembering to take their prescribed medication [3]. Health care professionals have an important role to play in supporting patients to self-manage, ideally working in partnership with patients [6].

A European standard for a minimum level of patient involvement was recently established with the aim to support a wider implementation of person-centered care (PCC) [7]. PCC is a health care approach where the patient’s subjective perception of illness and their preferences and values are the starting point for the care process. Partnership between patient and professional, as well as patient narratives and shared documentation, are considered key concepts in PCC. Within the narrative and examination, the patient’s need of care, prerequisites, resources, and obstacles are identified and documented together with the patient [8]. Attributes defining partnership vary in different publications, but shared decision making, shared knowledge, communication, and shared power are commonly mentioned. The consequences are described as empowerment of the patient and improved health outcome and health care utilization [9-11]. Patients appear to value other aspects of partnership than formal frames, appreciating proximity and receptive communication more than shared documentation and goal setting [12]. Using technological tools in health care may strengthen the potential for patient self-management, and the understanding and practice of partnership between patient and professional might change as a result [13].

### Objectives

Using an interactive information technology system requires interaction between patients and professionals, thus possibly affecting the patient-professional partnership. New roles for patients and professionals may be enabled. To date, there is limited research on how using technological tools in BP treatment affects the relationship between the patient and health care professional.

The objectives of this study were to explore the partnership between patients and health care professionals and further the roles of patient and professional when using an interactive web-based system for self-management of hypertension via the patient’s own mobile phone.

## Methods

### Study Design

**Figure 1:**
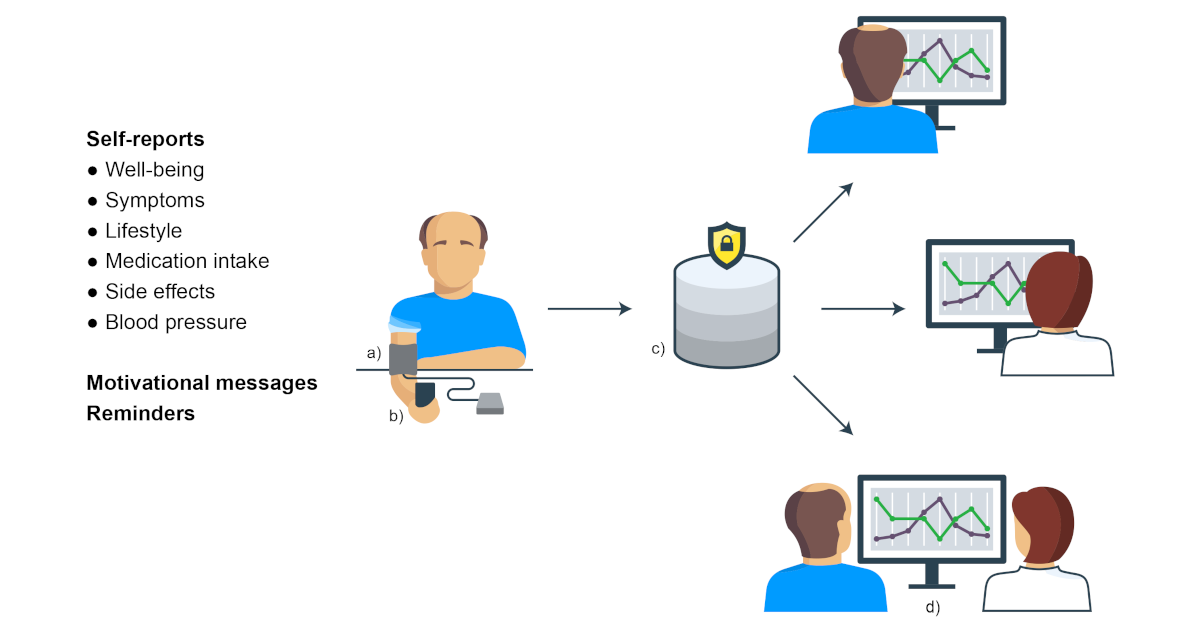
Overview of the interactive web-based communication system: (a) blood pressure device; (b) self-reports, reminders, and optional motivational messages via patient’s own mobile phone; (c) database where self-reports are saved; and (d) secure web portal available to patients and professionals for data visualization.

This study builds on a previously described interactive web-based communication system for self-management of hypertension called CQ (developed by Circadian Questions AB and referred to in this paper as “the system”). The system has been described in earlier publications [14-16], and an overview can be seen in Figure 1. During the planning, execution, and evaluation of the components of the system in the pilot project, the participating patients and professionals were actively involved [14-18]. The system was found to be relevant and easy to use [19], resulting in a significantly decreased BP for the participants (systolic BP –7 mm Hg and diastolic BP –4.9 mm Hg) [20]. Furthermore, use of the system was considered a resource for PCC and a more autonomous, knowledgeable, and active patient [21,22].

The system described in Figure 1 is now being tested in a randomized controlled trial (RCT; Person-Centeredness in Hypertension Management Using Information Technology [PERHIT]), including 900 patients with hypertension equally allocated to an intervention and a control group. The trial is conducted in primary care in 4 health care regions in southern Sweden. The aim of the trial is to lower BP in patients with hypertension in primary care. In addition, person-centeredness, patient self-reports such as daily life activities, and awareness of risk will be evaluated [23].

**Figure 2:**
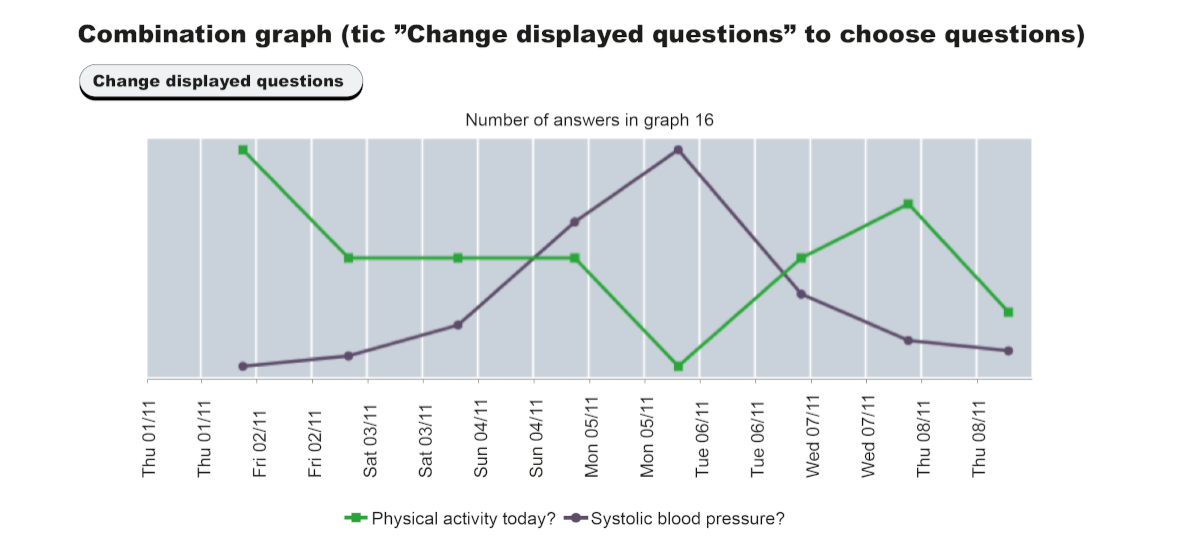
Graph showing correlation of physical activity with blood pressure, as shown to participants.

In short, the intervention consists of the following:

- Start-up meeting was scheduled with a nurse or physician at the local primary health care center (PHCC) where instructions were given about how to use the system at home, including measuring BP daily. Questions regarding side effects were selected according to the patients’ medication. Patients could choose to receive different relevant motivational messages on different days of the week. The messages were in the form of motivational questions and were intended to function as an inspiration for healthy choices (eg “Nice walk at lunch today?”). Patients also received a manual of the system and were advised to watch videos on BP measurement and how to enter data via their mobile phones.
- During 8 consecutive weeks, patients used the system at home and reported BP, symptoms, medication intake, side effects, lifestyle, and well-being. After log-in, patients and professionals had access to visualization of self-reported data in graphs via a secure web portal. All data was saved in a secure database, not in the mobile phones.
- Follow-up consultation was scheduled with a nurse or physician at the local PHCC after finishing the 8-week intervention. Professionals were encouraged to discuss graphs with patients. An example of a system graph is presented in Figure 2.
- Follow-up consultation was scheduled for 12 months after trial began.

Several interventions comprising mHealth (the use of mobile devices in health care) and hypertension have shown promising results [24-26]. However, the evidence is scarce, and several research studies have called for large RCTs with mHealth interventions that involve more patients for a longer time period [27-30].

In this qualitative study, we conducted focus group interviews with patients and professionals participating in PERHIT. The Consolidated Criteria for Reporting Qualitative Research (COREQ) checklist was used to ensure rigor in reporting the study design and conduction [31].

### Recruitment and Participants

Four PHCCs participating in PERHIT in different geographical and socioeconomic areas were strategically selected to reflect a broad socioeconomic area. Two of the PHCCs were located in midsize cities, one in a larger city suburb, and one in a smaller city. Patients and professionals were contacted by the staff at the PHCC to take part in focus group interviews.

At the time of the interviews, all patients had completed their 8-week intervention and attended the follow-up consultation with their nurse or physician. The time elapsed from the completion of the intervention to the interview varied between the patients from 1 week to 3 months (median 31 days). The inclusion criteria for the patients were the same as for the PERHIT study: aged older than 18 years, diagnosis of hypertension, treatment with at least one antihypertensive drug, and understanding of Swedish in order to be able to provide informed consent and make use of the system using the mobile phone for answering questions [23]. The inclusion criteria for the professionals were being a nurse or physician at the PHCC and having experience working with the PERHIT study.

Since only 2 to 4 professionals were involved in the study at each site, other professionals in the PERHIT study from nearby sites were also approached and asked to participate in the same interview. In total, professionals from 8 different PHCCs contributed to the study.

### Data Generation

Prior to the focus group interviews, 2 semistructured interview guides were developed by the research team, one for the patient groups and one for the health care professional groups. A test interview with mock patients was conducted prior to the first interview to evaluate the questions, resulting in some changes to the interview guide. After the first focus group interview with patients, it was obvious that a few questions needed to be rephrased. These were minor changes, and the material from the interview was still considered useful. No changes to the interview guides were made after that. Interview topics are presented in Textbox 1. Interviews began after introductions, small talk, and reiteration of the research goal [32].

**Interview topic and subtopic list.**

Hypertension and support:

- Current blood pressure treatment, drugs/lifestyle (patients)
- Perceptions of the most important treatment of blood pressure (professionals)
- Support in blood pressure treatment for patients from primary health care center in usual care

Patient-centered care and partnership:

- Perceptions of patient-centered care
- Perceptions of partnership or how to collaborate with health care professionals/patients, generally and specifically during the intervention
- Experiences from the follow-up consultation after 8 weeks
- Experiences of discussing patients in need of support in blood pressure treatment
- Perceptions of patients’ role in blood pressure treatment

Using the system and technology:

- Experiences of using the technology and how it was used during the 8-week intervention
- Perceptions of motivational messages
- How/if using the system has affected everyday life (patients)
- How/if using the system has affected working methods in blood pressure treatment (professionals)
- Experiences of using other technical systems for chronic disease in health care

Focus group interviews were held at the PHCCs from June 2019 to January 2020. A total of 22 patients participated in 4 focus group interviews, with 4 to 7 patients in each. Three focus group interviews, with 4 to 6 professionals each (n=15 total), were also conducted. No compensation was offered to the participants except for coffee and fruit during the interview. The duration of focus group interviews varied from 64 to 97 minutes and were held in Swedish. UA (first author) was the moderator of the focus group interviews. UB (second author), who is experienced in qualitative research, assisted and took notes. Prior to the focus group interview, UA had been in contact with the patients and professionals by telephone or email to set a date and time for the interview. No other relationship prior to the interview was established. At the interviews, researchers presented themselves with their occupation and as members of the research group conducting the RCT. Only the participants and researchers were present at the interviews.

### Data Analysis

The interviews were audiorecorded and transcribed verbatim. They were also videorecorded, with the purpose to serve as an aid for memory during the analysis phase. Thematic analysis according to Braun and Clarke [33] was used on the dataset, since it is a flexible method when performing qualitative analysis, allowing for both an inductive and deductive approach to the data [34].

The recordings of the interviews were listened through several times and the anonymized transcripts were checked against the recordings for errors by the first author (UA). During this phase, initial thoughts and ideas were noted. UA, UB, and KK (last author) read the transcripts repeatedly. UA created initial codes by systematically going through all the interview transcripts without a predefined coding frame. Interviews with patients and professionals were coded simultaneously using NVivo software (version 12, QSR International). The initial codes were compared and organized into common categories, which were discussed by UA, UB, and KK. Since we were interested in a specific aspect of the participants’ experiences—how using the system affected the experience of partnership between patients and professionals—we then used a deductive approach, inspired by previous research concerning the concept of partnership [8-11] and partnership and technology [13,35]. The initial codes were reviewed and arranged in preliminary themes and subthemes, focused on aspects of partnership. A narrative description of the preliminary themes and a thematic map were created and discussed by UA and KK. Themes were reviewed and checked against the datasets. In the process of defining and naming the themes, UA, KK, and AR collaborated and discussed until consensus was reached. A detailed description of each theme was developed, and informative names for the themes were established. To further visualize the themes, descriptive excerpts were identified.

### Ethical Considerations

The study was approved by the regional ethical review board in Lund (2017/311 and 2019/00036). Participants were given oral and written information about the study before they signed a consent form. All transcripts were anonymized to ensure confidentiality. The study was registered with ClinicalTrials.gov [NCT03554382].

## Results

### Study Sample

Characteristics of participating patients and professionals are presented in Tables 1 and 2.

**Table 1:** Characteristics of participating patients (n=22).

Characteristic		Value
Female, n (%)		8 (36)
Age (years), median (range)		65 (46-81)
**Age intervals (years), n (%)**		
	<50	2 (9)
	50-70	15 (68)
	>70	5 (23)
Country of birth, Sweden, n (%)		22 (100)
Years with hypertension^a^, median (range)		6 (1-39)
Number of hypertension drugs, median (range)		2 (1-4)
**Marital status, n (%)**		
	Married	15 (68)
	Unmarried	6 (27)
	Widowed	1 (5)
**Education level^b^, n (%)**		
	Up to high school	4 (18)
	High school	7 (32)
	University	9 (41)
**Employment status, n (%)**		
	Employed	10 (45)
	Retired	12 (55)

^a^Years with hypertension: missing 1 data point.

^b^Education level: missing 2 data points.

**Table 2:** Characteristics of participating health care professionals (n=15).

Characteristic		Value
Female, n (%)		10 (67)
Age (years), median (range)		55 (29-71)
**Age intervals (years), n (%)**		
	<40	3 (20)
	40-60	9 (60)
	>60	3 (20)
**Occupation, n (%)**		
	Assistant nurse	2 (13)
	Nurse	4 (27)
	District nurse	3 (20)
	Resident physician	1 (7)
	General practitioner	5 (33)
Years of experience working with patients with hypertension, median (range)		17 (4-30)

**Figure 3:**
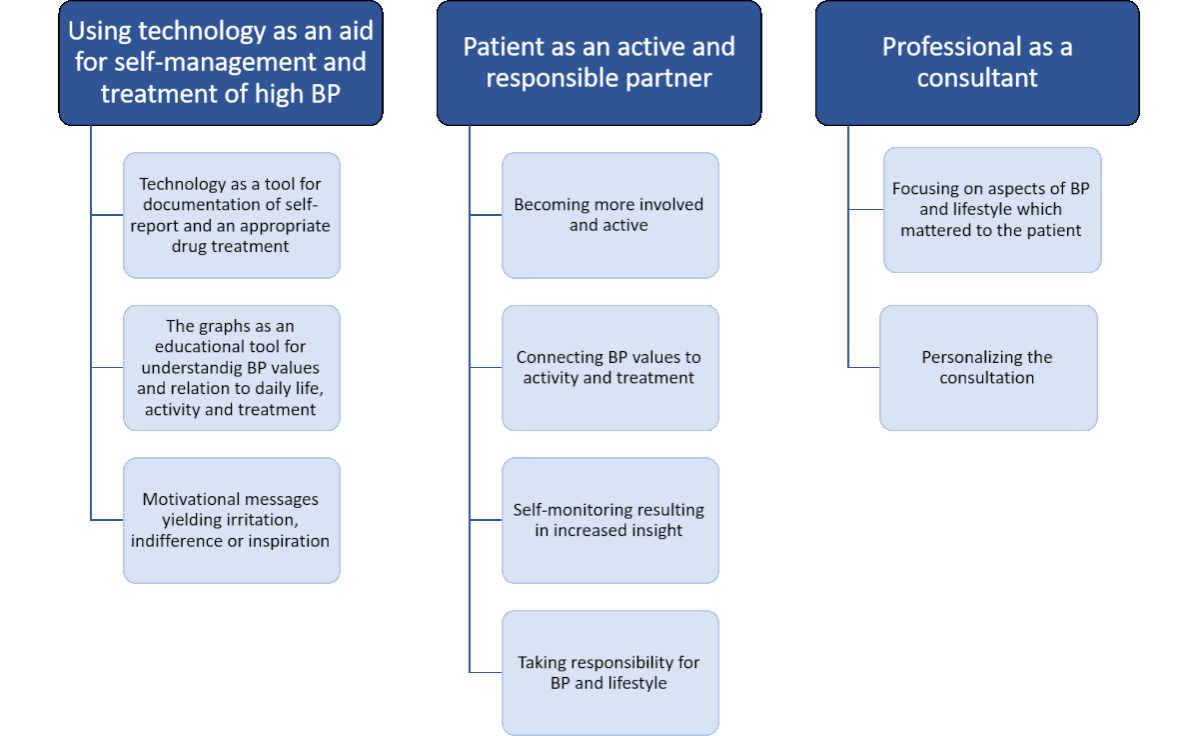
Overview of the themes and subthemes.

In the analysis of the focus group interviews, 3 actors were identified: the patient, the professional, and the technology. The roles of the different actors are described in 3 themes: using technology as an aid for self-management and treatment of high BP, professional as a consultant, and patient as active and responsible partner. An overview of the themes and subthemes is presented in a thematic map in Figure 3.

### Using Technology as an Aid for Self-Management and Treatment of High BP

#### Technology as a Tool for Documentation of Self-Reports and Appropriate Drug Treatment

The professionals considered the different components of the system to be helpful tools in the treatment of high BP. The documentation of the self-reports via the graphs made it possible to communicate more easily about the treatment. If a new drug was prescribed, the patient could follow the effect from day to day, thus becoming more aware of the BP treatment. It was considered a benefit that the patients monitored their BP at home instead of coming to the PHCC. During the intervention, some patients had contacted their nurse or physician when their BP was high, thus acting on high BP values.

Professionals viewed selected patients’ graphs during the 8-week intervention if the patient encountered problems adjusting the BP. If the BP was still too high, they contacted the patient and could adjust the drug treatment without the patient having to come to the PHCC. They believed this was educational for them as professionals as well, leading to increased understanding of the variation of BP.

And you could go in and see...see when they were running high, if something had happened, so to say, that day. If they were stressed or...if something...and if you saw that they were still running too high, so to say, you called and talked to them and said we need to adjust your medicine.

Health Care Professional 2 (HCP2)

For some of the patients, using the system brought a closer and more frequent contact with their prescribing physician. If they had altered their BP medication at the start of or during the intervention, they could with daily measurement report its effect on the BP.

But even with close, sort of, contact with M [the patient’s physician] which is...it’s been short telephone calls where you can...yeah, but he’s asked “How are you?” etc. Yeah, this is how it’s going now, and then we’ve been able to change it quickly.

Patient 13 (P13)

The professionals’ opinions about how feasible it would be to use the system as an integrated part of BP management differed. Not all were positive. Some experienced that it was too time-consuming and did not provide enough benefits to make it worthwhile.

#### Graphs as an Educational Tool for Understanding BP Values and Relation to Daily Life, Activity, and Treatment

During the follow-up consultation, graphs were used as an educational tool. Through the graphs, the patient could become aware of the normal variability of BP. They could also connect BP variations to physical activity, stress, or medication intake, for example, creating awareness of lifestyle and medication effect on BP. The patients contributed with their explanation of BP variation in relation to their daily activities.

But I myself had...in my head I kind of had the idea that now I want to see these graphs for these particular days and what I knew that I...had reported high then and also made a note of it, so to say, and it matched well. Yeah, I thought there was good correlation between these...

P22

Not all professionals viewed the graphs with the patients during the follow-up consultations, thus not using the system as intended. In those cases, the professionals expressed that the patients were passive during the follow-up consultation. The BP and lifestyle were not discussed, and instead the patients waited for the professional to introduce the next step in the study procedure. In these cases, the professionals had not adopted the instructions given by the research team regarding the intended use of the system.

#### Motivational Messages Yielding Irritation, Indifference, or Inspiration

The optional motivational messages included in the system, in the form of motivational questions, were meant to function as an inspiration for healthy choices. Opinions among the patients about the messages differed. Some patients perceived them as irritating since it was not possible to submit an answer. These patients had not been informed (and had not read the manual) about the intention with the messages to function as small reminders not requiring an answer. They thought a positive answer to the questions would generate further information. Others simply ignored the messages, since they disappeared in all the other incoming information in their mobile phones, such as text messages, emails and alerts. Other patients perceived the motivational messages as something positive, finding them an inspiration for healthy choices or considered them a small sign that someone cared about them.

### Patient as an Active and Responsible Partner

#### Becoming More Involved and Active

After using the system, patients were considered by the professionals to be more active in the consultation. They asked questions and wanted to discuss their BP values in relation to the documentation of their daily activities. The professional did not have to lead the conversation as they usually did.

Yeah, they were very serious; they had direct questions then, oh yeah, I saw that that day looked like this, what do you say about this, sort of...I didn’t have to ask that much; they had their questions for me.

HCP13

The patients considered themselves as more involved during the follow-up consultation, since they contributed with their knowledge about how they had felt and their health status. They considered themselves more prepared for the consultations and had thought about questions and what they wanted to discuss with the professional. They also believed this was recognized and confirmed by the professional, who was considered to be interested and attentive.

I’d say that you felt more like a participant, because I’d, like, been in this study and knew how I’d felt and how I, like...now it was...I could also offer something and contribute something.

P6

#### Connecting BP Values to Activity and Treatment

Using the system made the patients more aware of how their choices affected their BP and their health, and they reflected upon their days. Being able to measure the BP frequently gave insight into how different BP levels corresponded to daily activities.

But, you know, I’ve noticed right away when I’ve made that change there, I mean with the exercise and then also training with my dumbbells at home and stuff, that it’s had an effect; it has, you know.

P10

Not all patients logged in to the website and viewed their reported values in graphs during the intervention. Reasons for not logging in were that they were not aware of the possibility, they were not interested, or they chose to wait until the follow-up consultation. The patients who did view the graphs by themselves thought they were valuable and used them to relate activities or well-being to their BP values. Some patients who were not aware of the possibility to log in to the website kept notes by themselves, writing down the BP and what they had done and in some instances sharing their notes with their nurse or physician. Even if they did not view the graphs, they connected their daily BP value to how they felt or what they had done during the day.

#### Self-Monitoring Resulted in Increased Insight

By monitoring the BP and relating it to daily life, the patient became the expert on his or her BP. Some patients related that they got to know themselves and their bodies better. By daily monitoring, they could anticipate the BP value when measuring it in the evening. For example, after a stressful day, they expected a high BP value. They became aware of what affected the BP and what they could do about it. Their own responsibility for a successful treatment became clear to them.

Yeah, and it...it was a...yeah, it was actually a wake-up call too. That you could do something yourself; that you should do something yourself.

P6

#### Taking Responsibility for BP and Lifestyle

The patients regarded it as their responsibility to contact their physician or nurse when their BP was uncontrolled. They also considered it their responsibility to keep track of their BP and appreciated being able to check their BP at home.

So if I felt a little uncomfortable in my body and I went and checked my blood pressure and it was a total disaster, yeah, then I could sound the alarm earlier than if I hadn’t had a gauge. So in that way I feel safer now, I think.

P10

The professionals related that they saw an increased interest in self-monitoring of BP, even outside the settings of a study. They had noticed that some of their patients had bought BP monitors and used them at home. This was mostly considered positive, although the professionals also thought some patients measured and monitored excessively, which could lead to an increased workload for them and stress for the patient.

The patients considered diet and physical activity important regarding BP treatment. Participation in the study was a motivator for lifestyle changes such as increasing physical activity. They believed they had knowledge about the positive effects of exercise and a healthy diet prior to the study but had not taken it to heart before. Seeing the BP values every day became a reminder and encouragement to do something about the situation. The need to do well and be normal, to have a BP within the target values, was also a motivator for healthier choices. Some patients changed their dietary habits, cutting back on sweets, salt, and licorice. Others had thought about changing their habits but had not yet started.

I mean, I think, before I started this study I knew that exercise was the best, but even so...you heard it every time you came down and took your blood pressure and stuff, but yeah...you know, not much happens. But when I started this study I became much more focused; I thought I have to get this blood pressure down—I myself have to help too. So I’ve actually started exercising much, much more.

P6

### Professional as a Consultant

#### Focusing on Aspects of BP and Lifestyle That Mattered to the Patient

The professionals believed using the system contributed to more lifestyle-oriented conversations with the patients. Instead of only focusing on the effect of BP-lowering drugs, they talked about other aspects of high BP, such as how the patient’s lifestyle affected BP. The professionals considered the conversation to be more focused on the individual patient’s needs and resources than usual BP consultations. The patients said that they could discuss things that were important to them, that either they themselves or the professionals brought up. Some of the professionals expressed that they became more of a consultant for the patient than a lecturing nurse or physician. When the patients were more active during the follow-up consultations, possible lifestyle changes, which were significant for them, came to light and the discussion could focus on that on their terms.

And then maybe something turns up...one thing we can help with and work with, but then maybe we can calm down a little with the rest of...because it’s this that the patient’s a little interested in or feels like I have to...this...I can make a change here, and then we can help with that. Yeah, it was...it made it easier...to have that kind of discussion, I think.

HCP3

#### Personalizing the Consultation

By introducing the system to the patients and looking at the graphs together, the professionals related that they found out more about the patients and learned something new from them. One professional was surprised about how much the knowledge about BP differed between patients; some did not know about the risk of elevated BP or their own target BP. When this came to light, the discussion could be held in a more personalized way.

While using the graph as a visual tool, some of the professionals related that they learned new ways of talking about BP and lifestyle. Despite years of experience of talking to patients about BP, this consultation was considered more rewarding as it was more personal and relevant.

In some way I learned to teach people about blood pressure, which I actually hadn’t done before. I’ve seen so many blood pressure patients, but haven’t ever had the time to get into this particular person’s condition, kind of.

HCP13

Thus, not only could the patients gain new knowledge by using the system, the professionals could deepen their understanding of hypertension management.

### Partnership

As shown above, the system contributed to several attributes of partnership (see the code list from NVivo in Multimedia Appendix 1). Patients contributed with their knowledge about their health status and situations while professionals contributed with expert knowledge on BP, thus sharing knowledge. The professionals expressed that they learned new things using this working method. Both patients and professionals declared that the consultation was more equal as the patient was more prepared and knowledgeable, thus indicating shared power and shared collaborative decision making.

## Discussion

### Principal Findings

This study aimed to explore the partnership and roles of patients and professionals in hypertension management when using an interactive web-based system for self-management of hypertension. Focus group interviews with patients and professionals were conducted and analyzed using thematic analysis.

Three themes, on the technology, the patient, and the health care professional, are evident when using the interactive web-based system via mobile phone. The described themes represent one actor each. The system (the technology) is mainly a tool for documentation and sharing knowledge between patient and professional, thus affecting the partnership and how BP is communicated. By using the system, patients gained insight into how BP was affected by their lifestyle and became motivated to make healthier choices. As experts of their BP, they came well prepared to the follow-up consultation and were then able to take on a more active role. The professionals took a more secondary role during the follow-up consultation, controlling the conversation to a lesser extent. They were no longer the only holders of data and knowledge but instead became consultants and support to the patients, contributing with expert knowledge adjusted for the patients’ needs. Both patients and professionals described a consultation on more equal terms than usual, thus creating a base for a successful partnership. This was the case described by most of the participating professionals and patients but not shared by all.

### Comparison With Prior Work

Previous research has found that self-monitoring of BP enables activation of patients and motivates them to engage in lifestyle changes, favoring self-management [6,36]. By self-monitoring, the patient can provide the data that was previously produced by the health care professional at the clinical encounter. According to Shahaj et al [6], this might potentially challenge the dynamics between patient and professional, which is in line with the findings in this paper. Most of the patients in this study considered it their responsibility to check their BP regularly and adhere to the prescribed treatment. This was mainly viewed as something positive, but it could have potentially negative effects. If the patient using the system is not able to take on the responsibility, for example, not being able to interpret the BP values and acting on high values, the use of the system could be a burden. The system is intended to be used as a complement to the physical meeting and examination in usual care, and thus a patient not being able to use or interpret the system should not receive inferior care compared to treatment as normal. On the other hand, if the patient is able to take on the responsibility and self-manage effectively, the need for physical check-ups is diminished and contact with the health care professional can be managed over the phone or digitally in an effective way.

Wildevuur et al [13] studied how the partnership between patients and professionals is affected by the use of information and communication technology. They found that using information and communication technology in disease management requires an adjustment of the partnership through strengthened potential for self-management and shared analyzing of data. The health care system can be reorganized with new care pathways, where the data provided by the patient can serve as an initiative for treatment. Ultimately, it is the patients’ trust in technology and ability to self-manage that shapes the partnership with the professionals, provided that the professionals can adapt to the different needs of different patients.

In our interviews, opinions on using the system differed among professionals. Most of them found the system to be a helpful tool regarding hypertension management, inspiring new ways to talk about hypertension and working with the patient as an equal partner. Others were apprehensive about using it in clinical work since they found it too time-consuming. The professionals’ views about the role of technological tools in clinical work also differed; some did not believe it would bring any positive effects while others considered it an inevitable and possibly favorable part of their future working methods. A precondition for technology to enable effective PCC is, according to Wildevuur et al [35], that the technical solution is efficient for both patients and professionals and reduces the pressure on health care systems. In our study, the intervention technology is not integrated in the established health care technology, thus requiring the professionals to work in parallel systems, and this might cause problems. During the interviews, we found that the system was not used as intended in some instances despite a thorough introduction and a user manual. Some of the patients were not aware of the possibility of logging in to the web portal and viewing their reported values in graphs. This opportunity for visual feedback and insights of connections between BP and reported factors was therefore lost. Some of the professionals had not viewed the graphs together with the patients at the follow-up consultation after the 8-week intervention, thus disregarding a large part of the potential use of the system and an important kick-off for lifestyle changes during the rest of the 12 months. A lesson learned is that when introducing a new technical system, the professionals’ opinions and preferences about technology need to be acknowledged and considered. The professionals need to receive sufficient education on how to make use of the system in an optimal way and correctly instruct the patients on how to use it and what the benefits are for the patients in doing so. Implementation of a new way of working should bring benefits and not be considered a burden for the professionals. To establish a successful partnership, both the patient and professional need to be motivated about the new way of working.

The optional motivational messages in our study were received with mixed emotions by the patients. Previous interventions, which focused on text message–based lifestyle advice with the aim to lower BP, had shown small or insignificant positive results, indicating that motivational messages might be a part of a successful lifestyle intervention but are not sufficient on their own [37,38]. The irritation some of the patients described about the messages could be attributed to a lack of knowledge of the intention with the messages, highlighting the need for sufficient education of the health care professionals when conducting a study like this. That some patients were aware of the intention of the messages but chose to ignore them indicates that for a lifestyle intervention to be successful, some response or action is necessary. Otherwise, the message will disappear in the amount of information received daily.

Previous research has shown that follow-up consultations regarding hypertension are usually dominated by the professional and mainly focused on effect of drug treatment on BP [39]. As a contrast to this, during the follow-up consultations in this study, the focus was more on lifestyle and its relation to BP. The visualization of BP and lifestyle in graphs was considered valuable and contributed to the change of focus. The patients described that with insight gained by using the system at home and during the consultation came motivation to make lifestyle changes. This can lead to improved physical and psychosocial well-being beyond the effect on BP levels.

When used as intended, the system was found to be a resource for a person-centered approach in hypertension management. After implementing the system for 8 weeks, the patients could express their views and experiences of high BP. Both patients and professionals could contribute with knowledge during the follow-up consultation. The graphs could serve as documentation, shared by the patient and the professional.

To further analyze the potential benefit of using this system, future studies could focus on testing in other clinical or cultural settings such as hospital clinics with outpatient care or in other countries with a more diverse population. As reported by Samkange-Zeeb et al [40], it is important to consider migration background and language competency to make information and services via the internet accessible in diverse groups.

### Strengths and Limitations of the Study

A strength of this study is that it builds on previous work and confirms results found in the pilot project regarding the potential of the system to support patient self-management. By including experiences from both patients and professionals in the study, a more comprehensive dataset was obtained. Both perspectives must be identified before implementation in clinical practice.

This study has some limitations. During recruitment for the focus group interviews, we aimed to put together groups with a diversity of men and women from different socioeconomic and cultural backgrounds and in different age groups. We therefore approached PHCCs in different socioeconomic areas. The number of eligible patients, with the diversity above, per PHCC was limited and not all proposed patients agreed to participate. We therefore had to make some compromises in selection of patients. One included health care center in a multicultural area unfortunately had to be excluded from the trial due to language problems and following methodological errors. Also, an inclusion criterion was to understand Swedish. Consequently, we did not achieve a diversity in terms of ethnicity and cultural background. The participants were comparable with the Swedish hypertension population in terms of age, but the sex distribution differed, with a majority of men in our study [41]. As always in studies like this, there is a risk for selection bias in the recruitment of patients. The patients who are already aware of their health status and motivated to treat their condition may have chosen to participate in the trial to a higher degree.

### Conclusion

Using technology for strengthening patients’ potential for self-management has the possibility to change the relationship between patients and professionals. The patients perceived themselves as more active and motivated in their BP treatment. When using the system as intended, the professionals experienced it as a resource for communication regarding BP and lifestyle. Both patients and professionals described a consultation on more equal grounds, laying the foundation for a constructive partnership.

To realize the potential in a system like this, health care professionals need to be motivated and interested in new approaches in management of chronic conditions. Integration of the technology in the existing technical system is essential. Health care professionals also need to receive a thorough introduction so that they, in turn, can properly instruct and motivate patients to use the system and read the manual. If this is not achievable, introduction of a new technical solution may instead increase workload and become a burden in chronic condition management both for professionals and patients.

## Appendix

Multimedia Appendix 1 Codes for focus group interview with patients.

## Abbreviations

BP: blood pressure
COREQ: Consolidated Criteria for Reporting Qualitative Research
mHealth: mobile health
PCC: person-centered care
PERHIT: Person-Centeredness in Hypertension Management Using Information Technology
PHCC: primary health care center
RCT: randomized controlled trial
