# Supplementary material for: Patients and Professionals as Partners in Hypertension Care: Qualitative Substudy of a Randomized Controlled Trial Using an Interactive Web-Based System Via Mobile Phone
Source: J Med Internet Res. 2021 Jun 3;23(6):e26143. doi: 10.2196/26143 (PMC8212623; doi:10.2196/26143)
Supplement: Multimedia Appendix 1 [file jmir_v23i6e26143_app1.pdf]

Code list from Nvivo

(translated from Swedish)

Table 1. Codes for focus group interviews with patients

Codename	Files[a]	References[b]
Communication	0	0
The patient more active	2	8
A more personal consultation	3	7
Patient autonomy	0	0
Expert in own blood pressure	4	18
Patient participation	0	0
Connecting BP values to activities	4	23
Taking responsibility for a healthy lifestyle	4	35
Taking responsibility for own BP	0	0
Contacts health care when values are abnormal	2	5
Taking responsibility for contact with health care	3	5
Taking responsibility for BP monitoring	4	15
Using the graphs on your own	3	7
Perceptions about PCC and partnership	0	0
Perceptions of PCC	4	14
Thoughts about what partnership can be	1	1
Allowed to take time and space	4	10
Cooperation about BP treatment	0	0
Focus on BP	2	2
Hard to motivate oneself	1	2
Shared responsibility form health care	1	4
Wants to be called	1	2
Established relationship with trust and professionality	4	29
Feedback from health care is needed	2	8
Individualized care	1	1
Information as support	1	1
Shared knowledge	0	0
Using the graphs as support in the consultation	3	11
Cooperating with professionals	4	10
Recieved information from professional	1	1

Table 2. Codes for focus group interviews with health care professionals

**Codename**	**Files[a]**	**References[b]**
Communication	0	0
Individualized consultation	2	8
More equal consultation	1	3
More holisitic consultation	3	7
The patient more active	2	5
The patient more passive	1	1
IT-literacy	3	9
Patient autonomy	0	0
Expert in own BP	3	7
Patient participation	0	0
Connecting BP values to activity	1	2
Takes responsibility for a healthy lifestyle	2	5
Doubts the patient's capability for lifestyle changes	3	8
The patient more involved	3	5
The patients take responsibility for high BP	0	0
Contact health care when values are abnormal	1	3
Excessive measuring is negative	3	7
Takes responsibility through BP monitoring	3	6
Perceptions of PCC and partnership	0	0
Thoughts about partnership	3	19
Thoughts about PCC	3	23
Shared knowledge	0	0
Cooperation with health care professionals	2	3
Health care based on the patient's preconditions	1	3
The graphs as a support in the consultation	3	12
The patient's knowledge is evident	2	3
Tool for the professionals	3	11

[a]Each focus group interview is represented by a file.

[b]References represents the number of data extracts coded to each codename.

Abbreviations

BP: Blood Pressure
PCC: Person-centered care

This is a Multimedia Appendix to a full manuscript published in the J Med Internet Res. For full copyright and citation information see http://dx.doi.org/10.2196/jmir.26143
